# Supplementary material for: Molecular Evolution of SNAREs in Vitis vinifera and Expression Analysis under Phytohormones and Abiotic Stress
Source: Int J Mol Sci. 2024 May 30;25(11):5984. doi: 10.3390/ijms25115984 (PMC11173047; doi:10.3390/ijms25115984)
Supplement: Supplementary file 1 [file ijms-25-05984-s001.zip › Table S1.pdf]

Gene	Forward primer (5′→3′)	Reverse primer (5′→3′)
VvSNARE1	GTGGCTCGTGGGACTGTGATTC	TCTGAAGGCACTGAAAGGCAATGG
VvSNARE2	GTACGGATTGGAGTGGGACAAAGAC	CAAGGGTGAGGGTGTGTATGGAAAC
VvSNARE3	ATCAACAGCGGCAAACTCTCCTAC	GTTCCTGCTCATCCTCTTCGACATG
VvSNARE4	CAACAGCACAGGCAAAATCCCAAC	CCAACAGCAAGCAGGTCAGAGATG
VvSNARE5	ATGGGGTCGTTGATTGGAGTTGTG	AAAGGGAGTGCTGTTCGTGTGATG
VvSNARE6	GAGAGGAGAGGAGAGAGGGATTGC	CAGAGGCGGGCATGGTCAAATAC
VvSNARE7	AGGAACCCAGTGAGGAAGTCGTAG	AGCCTCATGCCTCTCCTGATTCTC
VvSNARE8	GTCACGAATGGGCTGAGGAAGAAG	ACCGTCTCCTTGTACTCCGTCATC
VvSNARE9	TGGCTACCAGGTCTGCTGCTAG	TGCTGCTGCTGCTGCTGTATC
VvSNARE10	TGGAAGAAGGTGGTGGCTCTGAG	CCCAAAGGTTGAGCGTTGAAAGAC
VvSNARE11	TCAAGGGTTCTGAGCCAGCAAATG	CAACTCCAAGCGATCACCTCTGTC
VvSNARE12	ATTGAGAAGGTTCTTGACCGTGGTG	AATCTTGTGCCTGTGAGCGAAGG
VvSNARE13	ACAGTTGCTTCTCATAAGGCGACAG	AAGCCCAACTCCTCCAATTCTTTCC
VvSNARE14	TCCTGCCCAACCTCATTTCCAAAC	AGCCAGACAACCGTATTCCAGATTG
VvSNARE15	GGAACGATGGTATTGGCGGAGTAC	GGTGTGGTGATCGCAGTTGTAGG
VvSNARE16	GGTGGGTCAGCAGTCTTTGATCTAC	TGAAGGCATTGAGCGGCGATG
VvSNARE17	ATCGCCGTCGCTTGTTTCACTC	GCCAGGACTGTCAGAGGAATGTAAG
VvSNARE18	GCTCGGTTGCTAGAGGAAGTTGTC	CTGCCATGCTCCCATCTGGTATTG
VvSNARE19	GGACCGTGGTGCTTTCTGAGTTC	CTGCGAGAAACAGAGCCTGGAATC
VvSNARE20	AACTCCCTGCCACCAACAACAAG	CAACAGCCTCAACAGCAACAACAC
VvSNARE21	AAGGCTTCTTTGGACAAGGCACTAG	TCTGGATCGTGAGAGGCTTATGGAG
VvSNARE22	GCGTTCTCTTGCTACAGACCTTCAG	CATCTCCAAGTCAACCCCGTCATG
VvSNARE23	AGAAGGCAGCAAGCATTACAAGGG	AGCAGACACCAGCAAAGAAACACTC
VvSNARE24	TCCAAAGGGCCAAGAAGCTACAAAG	CTTGAGCACTGCCACCACTATGATC
VvSNARE25	TCGTCTTTACGCCGTCGTTGAAG	ACTTAGGGACCTCCTCCAGCAATC
VvSNARE26	GCCGCAAGTGGACCTGTTACG	GGTGGAGGTGGAGGTGGAGATG
VvSNARE27	TTGAAGCTGATTGAGAGGCGGAAC	GTACAAGACCACGACTGACACAACC
VvSNARE28	GCTCGTGTTACTGATGGGCTTCC	TGACATTCTTGAGGGCTCATTCTGG
VvSNARE29	CTCCTCCTTGGGCCAATGAATCTTC	GTTGTTGTTGCTGCTGCTGCTG
VvSNARE30	CAGATGGCCTCACCCTTCTTTGC	CATCATTCATGGCGTAGGCTGGAG
VvSNARE31	TCGCTTTTGTGCTTGTTGCTTCTC	TCATTCCAGCCAACCACATTGAGG
VvSNARE32	GGGCAGCACCTGAACGACATC	GCTCTTCTGGTAGTTCCTCGCAATC
VvSNARE33	TGGAGACCAATGGAGAAGAAGCATC	ATGAAGAGAAACCCAGGTGCTTGG
VvSNARE34	TGCCAAAGTTCAGCCTCCCAAAG	GCTTATCCACCTGCTTCTCAACCTC
VvSNARE35	CACCTCATGTCCACCCATATTCTGC	TGCTGCTGCTATTTCGCTGGATG
VvSNARE36	ACTGTGACTGAGCCACCTCCTTG	GCCAACTTGAACTCCATTTGACGAC
VvSNARE37	ACCTTCCAACAGCAGCAAGTACAC	CATCCTTCACACGCTCAAGAAACAC
VvSNARE38	GGATGACAACTACCCAGTGCGAAG	GGTTGAGTGACATCTGCCTGAGC
VvSNARE39	TCTCTTGCTACCGACCTCCAGAAC	CCCCATCCTGACCTTCCTTTTGC
VvSNARE40	GACACGCTGCATGGAGTAGATGAC	AGGCAATGACAACAACGAGGACTG
VvSNARE41	CCGACAACACTAGCGCCATCTC	TGCAAAGGAACGTAAGCCCATCAG
VvSNARE42	TGCTAGTGCCACCAACATCAAGTAC	TTTGCCCCATCTGCCAAGTTCC
VvSNARE43	CCGTGGCCTGCTTGGTATATTACTG	GATCGCCGCCTCCTGATTGAAC

VvSNARE44	GGCAACAAAGAGGCGGGAAGAG	AGCGAATTGACAAGGCGATAGAAGG
VvSNARE45	AATAGTTGGAAGGGTAAGCGATGGG	ATCTTTGAAGGTGACAAGGCTCCTC
VvSNARE46	CACCAACAACGCCAGTCTCTCC	GACATAGCCGTCAGGATCTTCTTGC
VvSNARE47	CGAATGAGGATGATTCTCCGACCAC	ACTCCCAGAGCCACCGTGAAC
VvSNARE48	GTGGCGGTAACGGTGACGATG	ACAACACCCACAAGCAGCAGAC
VvSNARE49	AATGGCTTCTTCAGACTCGTGGATG	ATCGCAGATGCATGACGTTGGG
VvSNARE50	TGCCGATGCCATGAGCAGAAC	CTCGTTGACCGCCAATGCAATATG
VvSNARE51	CGTAGCTCAGTGGTTAGTGGATTGG	CGCCTTCTGTCCTGTTATGGTGTAG
VvSNARE52	TGCCGATGCCATGAGCAGAAC	CTCGTTGACCGCCAATGCAATATG
VvSNARE37-EGFP	TTTGGAGAGGACACGCTCGAGATGAGTCA GAAAGGCTTAATATATAGCTTT	GCCCTTGCTCACCATGAATTCACATTTGAAACCCTTA CAGGCTATAA
VvSNARE44-EGFP	TTTGGAGAGGACACGCTCGAGATGAGCTTTGAA GATCTTGAATGGGG	GCCCTTGCTCACCATGAATTCGTTCATGTTTCTCGGC ATCACC
VvSNARE46-EGFP	TTTGGAGAGGACACGCTCGAGATGAGCCAGGTAT TCGAAGGATA	GCCCTTGCTCACCATGAATTCATGAGCCAGCTTGAA ATAAAGGA
