# Supplementary material for: Molecular Evolution of SNAREs in Vitis vinifera and Expression Analysis under Phytohormones and Abiotic Stress
Source: Int J Mol Sci. 2024 May 30;25(11):5984. doi: 10.3390/ijms25115984 (PMC11173047; doi:10.3390/ijms25115984)
Supplement: Supplementary file 1 [file ijms-25-05984-s001.zip › Table S2.pdf]

Gene	Gene accession No.	Chromosome location	Full length/bp	Length of CDS/bp	Amino acid	Molecular weight/kD	pI	Formula
VvSNARE1	VIT_201s0011g01660	1	3422	666	221	24950.95	8.45	$C_{1115}H_{1774}N_{300}O_{322}S_{13}$
VvSNARE2	VIT_201s0150g00430	1	14007	795	264	28858.66	9.46	$C_{1322}H_{2083}N_{345}O_{362}S_{8}$
VvSNARE3	VIT_201s0113g00540	1	22854	663	220	24901.83	8.41	$C_{1084}H_{1811}N_{309}O_{336}S_{11}$
VvSNARE4	VIT_202s0012g01850	2	10254	825	274	30166.32	5.75	$C_{1328}H_{2176}N_{374}O_{418}S_{3}$
VvSNARE5	VIT_202s0025g04990	2	7639	882	293	32431.16	6.22	$C_{1520}H_{2339}N_{365}O_{397}S_{11}$
VvSNARE6	VIT_202s0012g01630	2	2580	765	254	28279.28	8.31	$C_{1312}H_{2034}N_{318}O_{350}S_{13}$
VvSNARE7	VIT_203s0038g04720	3	900	900	299	34179.20	5.30	$C_{1487}H_{2440}N_{416}O_{470}S_{16}$
VvSNARE8	VIT_204s0008g02770	4	936	936	311	35413.85	6.07	$C_{1543}H_{2531}N_{437}O_{475}S_{19}$
VvSNARE9	VIT_204s0008g04350	4	3662	1023	340	38119.10	9.22	$C_{1669}H_{2684}N_{478}O_{524}S_{9}$
VvSNARE10	VIT_204s0008g05920	4	10978	663	220	24909.39	8.63	$C_{1095}H_{1775}N_{313}O_{337}S_{6}$
VvSNARE11	VIT_204s0008g05650	4	4712	663	220	25040.88	9.48	$C_{1110}H_{1776}N_{322}O_{318}S_{10}$
VvSNARE12	VIT_205s0136g00290	5	1165	486	160	18147.59	9.39	$C_{807}H_{1338}N_{224}O_{224}S_{12}$
VvSNARE13	VIT_205s0094g00860	5	4674	744	247	27994.84	4.95	$C_{1226}H_{1973}N_{337}O_{388}S_{11}$
VvSNARE14	VIT_205s0020g03160	5	7461	1320	439	48700.86	6.84	$C_{2252}H_{3504}N_{558}O_{614}S_{15}$
VvSNARE15	VIT_205s0020g01430	5	3185	666	221	25180.27	8.98	$C_{1130}H_{1793}N_{301}O_{323}S_{13}$
VvSNARE16	VIT_205s0136g00285	5	10180	339	112	12249.84	7.68	$C_{550}H_{840}N_{146}O_{164}S_{4}$
VvSNARE17	VIT_205s0020g02660	5	15681	825	274	29789.60	6.11	$C_{1405}H_{2226}N_{330}O_{362}S_{8}$
VvSNARE18	VIT_206s0004g03090	6	5100	798	265	30059.32	5.34	$C_{1315}H_{2147}N_{367}O_{415}S_{10}$
VvSNARE19	VIT_206s0080g00800	6	9406	663	220	24899.14	9.20	$C_{1109}H_{1782}N_{310}O_{310}S_{15}$
VvSNARE20	VIT_207s0005g05790	7	6034	660	219	24602.71	8.82	$C_{1100}H_{1770}N_{294}O_{317}S_{13}$
VvSNARE21	VIT_207s0104g00260	7	9470	816	271	29509.26	9.16	$C_{1391}H_{2194}N_{330}O_{355}S_{9}$
VvSNARE22	VIT_207s0005g01000	7	3476	957	318	35836.00	7.72	$C_{1554}H_{2561}N_{451}O_{489}S_{14}$
VvSNARE23	VIT_207s0005g06610	7	29805	1170	389	42583.16	8.47	$C_{1972}H_{2998}N_{496}O_{536}S_{11}$
VvSNARE24	VIT_208s0032g00440	8	22401	924	307	34663.76	6.95	$C_{1506}H_{2508}N_{442}O_{471}S_{10}$
VvSNARE25	VIT_208s0040g02130	8	11078	798	265	29760.84	5.14	$C_{1295}H_{2121}N_{367}O_{415}S_{9}$
VvSNARE26	VIT_208s0032g01150	8	975	975	324	36291.84	6.69	$C_{1572}H_{2553}N_{463}O_{506}S_{8}$
VvSNARE27	VIT_208s0056g01310	8	5252	675	224	25931.72	9.44	$C_{1138}H_{1842}N_{334}O_{340}S_{9}$
VvSNARE28	VIT_209s0002g00780	9	2761	657	218	25463.51	9.38	$C_{1150}H_{1830}N_{314}O_{324}S_{7}$
VvSNARE29	VIT_211s0016g04370	11	3973	1017	338	37618.57	9.08	$C_{1639}H_{2652}N_{468}O_{522}S_{11}$
VvSNARE30	VIT_211s0016g05850	11	1392	327	108	12519.09	7.06	$C_{560}H_{839}N_{159}O_{160}S_{5}$
VvSNARE31	VIT_212s0035g02270	12	1769	963	320	36540.04	7.03	$C_{1717}H_{2627}N_{415}O_{440}S_{13}$
VvSNARE32	VIT_212s0059g02060	12	918	918	305	34535.29	6.12	$C_{1504}H_{2436}N_{434}O_{470}S_{13}$
VvSNARE33	VIT_212s0035g02200	12	913	441	146	16703.40	6.28	$C_{779}H_{1184}N_{188}O_{210}S_{5}$
VvSNARE34	VIT_212s0059g02340	12	3140	915	304	34676.11	8.61	$C_{1513}H_{2528}N_{432}O_{468}S_{13}$
VvSNARE35	VIT_213s0019g03240	13	4948	930	309	35562.37	6.09	$C_{1556}H_{2518}N_{450}O_{484}S_{9}$
VvSNARE36	VIT_213s0067g00280	13	18301	1188	395	43975.27	8.77	$C_{1940}H_{3103}N_{549}O_{584}S_{16}$
VvSNARE37	VIT_213s0101g00410	13	7304	720	239	27275.38	6.23	$C_{1221}H_{1920}N_{328}O_{356}S_{12}$
VvSNARE38	VIT_214s0060g00090	14	4042	630	209	23753.13	6.95	$C_{1053}H_{1654}N_{290}O_{314}S_{11}$
VvSNARE39	VIT_214s0060g01540	14	6580	984	327	36412.66	8.87	$C_{1575}H_{2621}N_{455}O_{504}S_{13}$
VvSNARE40	VIT_214s0108g01200	14	4633	666	221	24771.45	9.10	$C_{1057}H_{1785}N_{319}O_{339}S_{12}$
VvSNARE41	VIT_215s0045g01290	15	288	288	95	10904.83	9.02	$C_{493}H_{788}N_{138}O_{131}S_{5}$
VvSNARE42	VIT_215s0048g01770	15	1461	825	274	30304.89	9.03	$C_{1423}H_{2223}N_{347}O_{366}S_{8}$

VvSNARE43	VIT_215s0048g00540	15	9576	849	282	31697.41	9.12	$C_{1391}H_{2247}N_{399}O_{418}S_{14}$
VvSNARE44	VIT_216s0039g01850	16	4273	849	282	31654.87	6.27	$C_{1383}H_{2253}N_{399}O_{437}S_{6}$
VvSNARE45	VIT_216s0050g00060	16	858	540	179	20863.99	7.64	$C_{939}H_{1452}N_{252}O_{267}S_{10}$
VvSNARE46	VIT_217s0000g06580	17	12744	666	221	24840.68	9.61	$C_{1070}H_{1800}N_{322}O_{332}S_{11}$
VvSNARE47	VIT_218s0072g01050	18	11439	840	279	31486.26	9.58	$C_{1469}H_{2295}N_{365}O_{379}S_{11}$
VvSNARE48	VIT_218s0001g03830	18	6217	1002	333	35493.05	9.40	$C_{1633}H_{2556}N_{422}O_{454}S_{4}$
VvSNARE49	VIT_219s0090g01300	19	2421	798	265	29587.37	8.93	$C_{1293}H_{2135}N_{369}O_{389}S_{16}$
VvSNARE50	VIT_200s0227g00170	UN	833	507	168	18872.07	7.75	$C_{819}H_{1382}N_{236}O_{249}S_{11}$
VvSNARE51	VIT_200s0404g00100	UN	915	915	304	34216.97	5.87	$C_{1483}H_{2422}N_{420}O_{475}S_{15}$
VvSNARE52	VIT_200s0582g00030	UN	796	471	156	17416.38	7.74	$C_{754}H_{1280}N_{218}O_{231}S_{10}$
