# Supplementary figures and images for: Molecular Evolution of SNAREs in Vitis vinifera and Expression Analysis under Phytohormones and Abiotic Stress
Source: Int J Mol Sci. 2024 May 30;25(11):5984. doi: 10.3390/ijms25115984 (PMC11173047; doi:10.3390/ijms25115984)

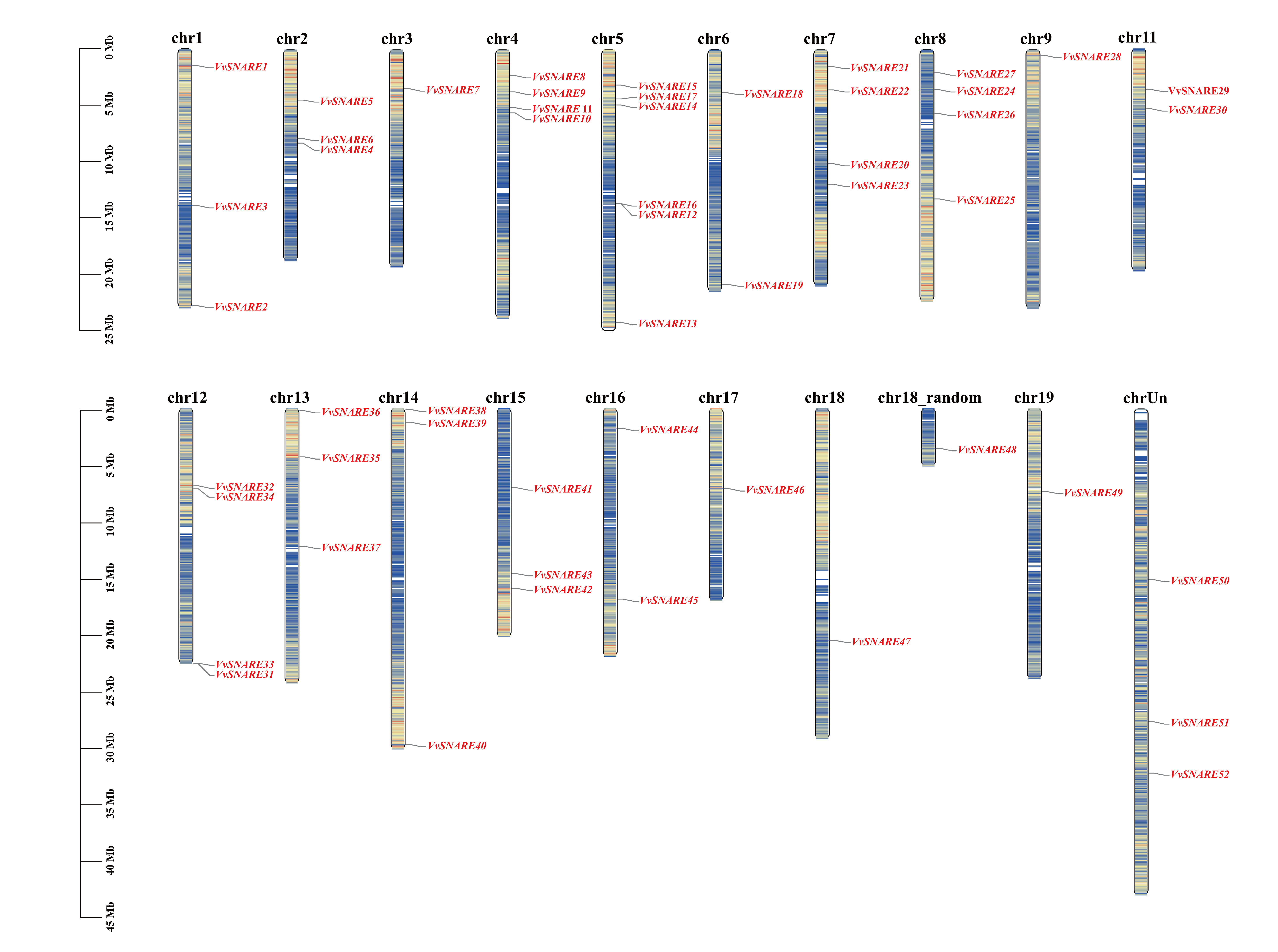

Supplement: Supplementary file 1 [file ijms-25-05984-s001.zip › Figure S1..tif]

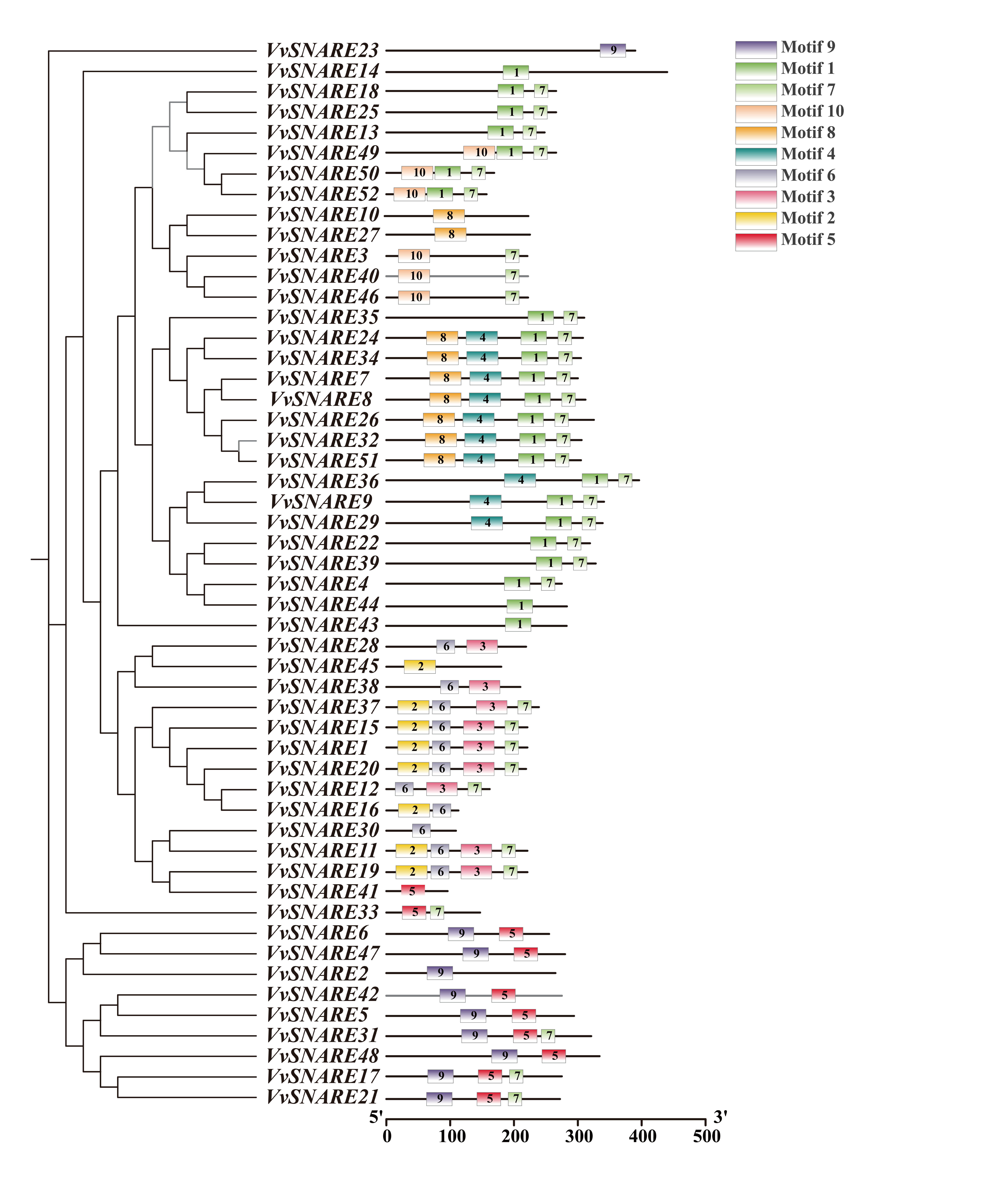

Supplement: Supplementary file 1 [file ijms-25-05984-s001.zip › Figure S2.tif]

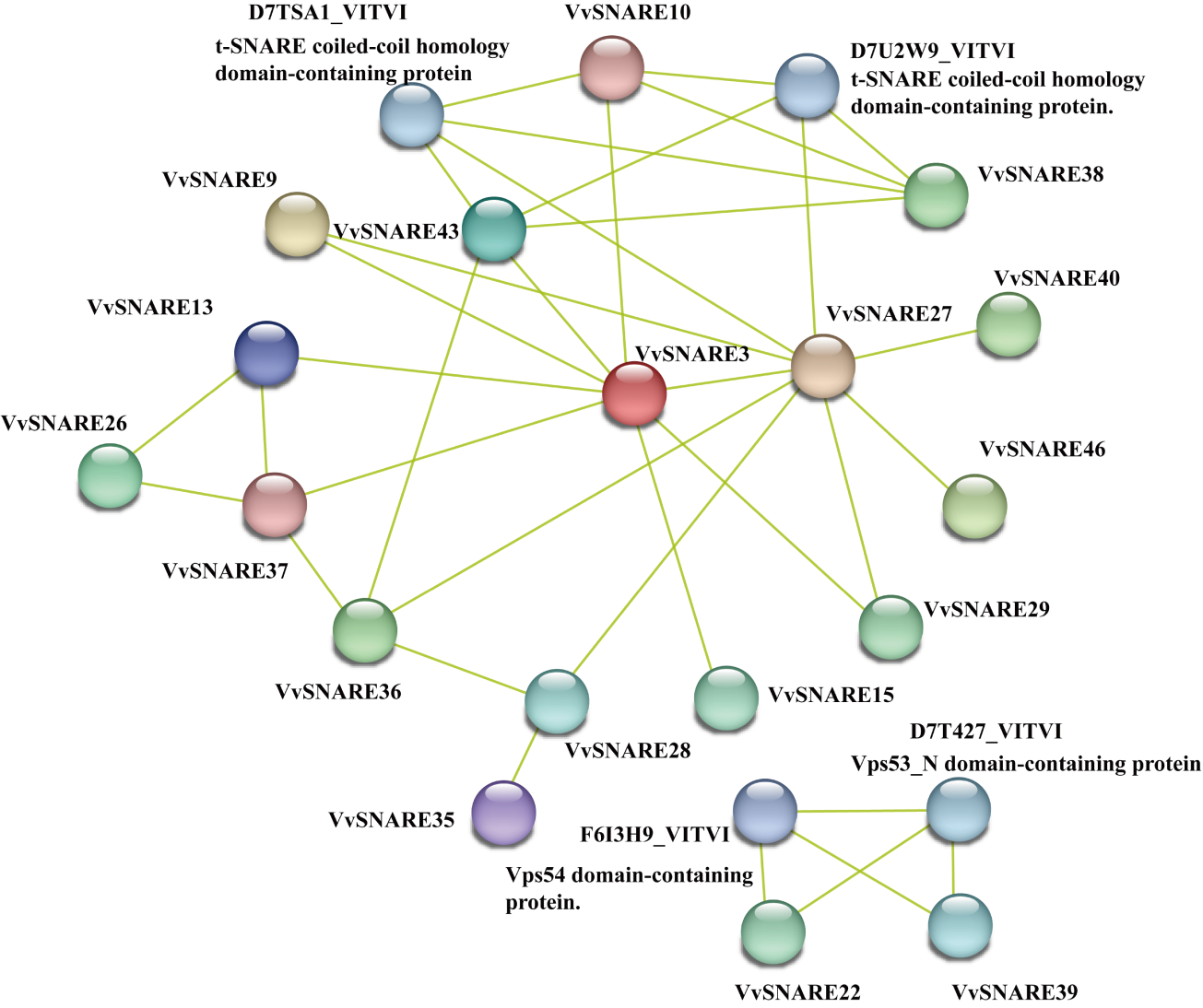

Supplement: Supplementary file 1 [file ijms-25-05984-s001.zip › Figure S3 .tif]

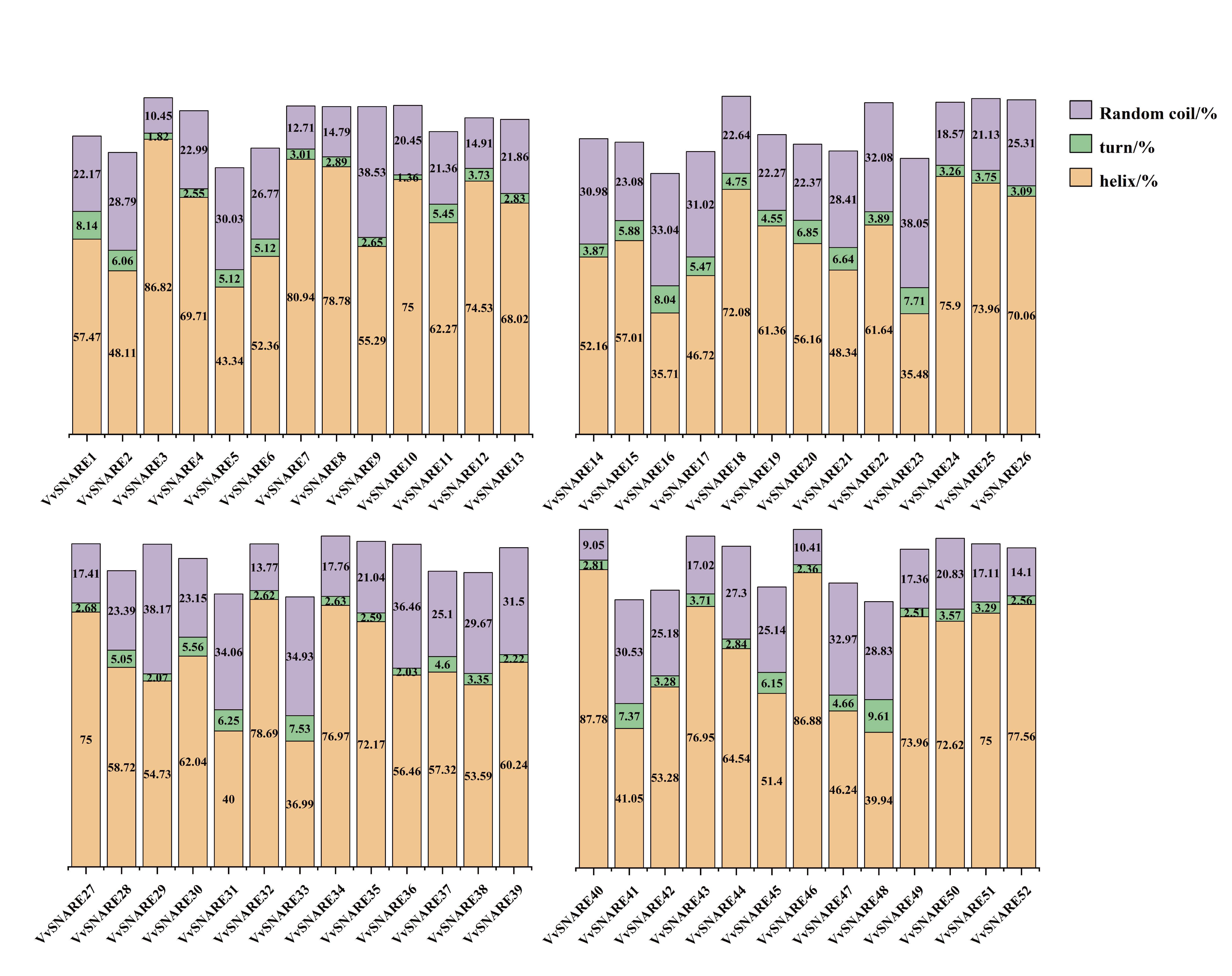

Supplement: Supplementary file 1 [file ijms-25-05984-s001.zip › Figure S4.tif]

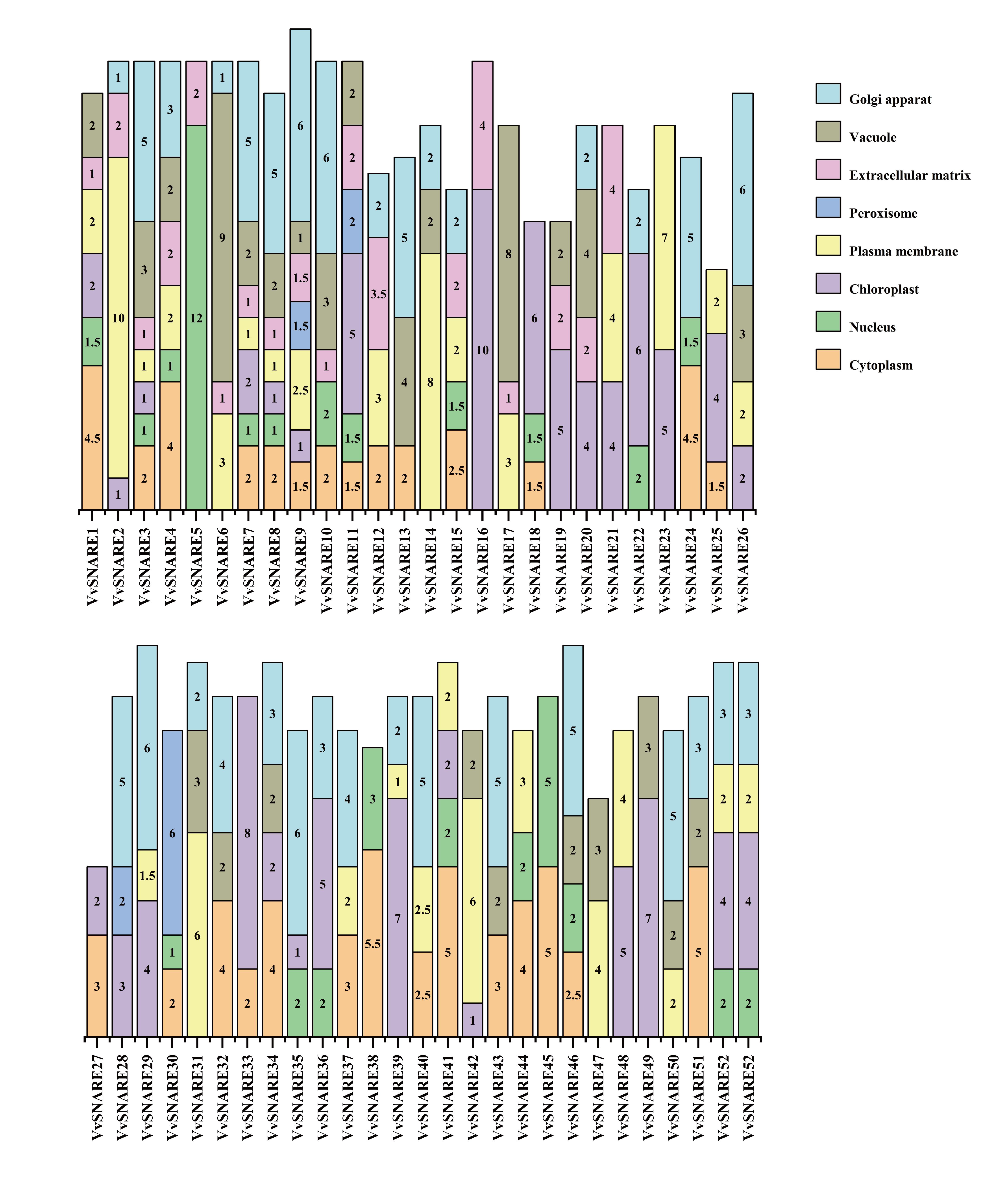

Supplement: Supplementary file 1 [file ijms-25-05984-s001.zip › Figure S5.tif]

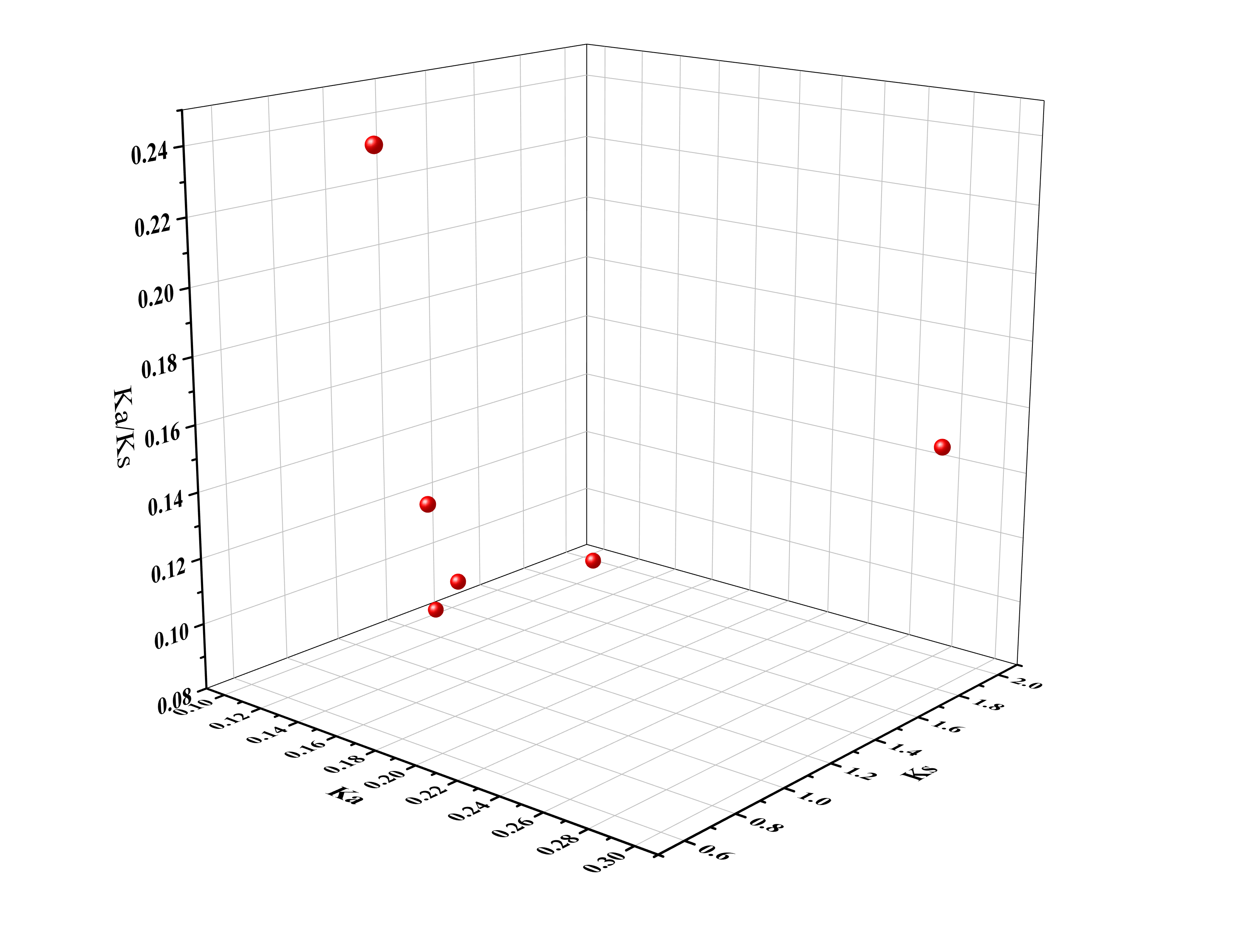

Supplement: Supplementary file 1 [file ijms-25-05984-s001.zip › Figure S6.tif]
